# MIDLIFE NEIGHBORHOOD SOCIOECONOMIC STATUS AND COGNITIVE FUNCTION IN LATE LIFE: DIFFERENCES BY RACE

**DOI:** 10.1093/geroni/igae098.1501

**Published:** 2024-12-31

**Authors:** Greta Jianjia Cheng, Jeanine Buchanich, Tiffany Gary-Webb, Christina Mair, C Shaaban, Andrea Rosso

**Affiliations:** University of Pittsburgh, Pittsburgh, Pennsylvania, United States; University of Pittsburgh, Pittsburgh, Pennsylvania, United States; University of Pittsburgh, Pittsburgh, Pennsylvania, United States; University of Pittsburgh, Pittsburgh, Pennsylvania, United States; University of Pittsburgh, Pittsburgh, Pennsylvania, United States; University of Pittsburgh, Pittsburgh, Pennsylvania, United States

## Abstract

Evidence regarding the role of neighborhood socioeconomic status (NSES) as an upstream determinant of cognitive outcomes has lacked a life-course perspective and has relied on time-invariant NSES estimates. Compared to late-life, midlife NSES may be more relevant to cognitive outcomes, and associations with cognition may vary by race, reflecting the potential impact of structural racism. We examined racial differences in associations between midlife NSES and changes in cognitive function among older adults aged 70+. We included 341 (37% Black and 63% White) participants from the Pittsburgh site of Health, Aging, and Body Composition Study. Cognitive function was assessed repeatedly by Modified Mini-Mental State Examination (range: 80-100) throughout a 13-year follow-up. LexisNexis addresses from 1976-1985 were geocoded and linked to 1980 Census estimates (education, occupation, employment rate, household income, and home value). Midlife NSES (aged 49 to 58 years) for census tracts was calculated as the sum of z-scores of five estimates (range: -10.4 to 9.1, SD: 4.3). In linear mixed-effects models adjusted for census-tract and individual-level characteristics, midlife NSES was associated with baseline cognitive function among Black (β: 0.56, 95% CI: 0.14, 0.98), but not among White participants (β: -0.05, 95% CI: -0.31, 0.21) (p-value for interaction: 0.02). There were no observed associations between midlife NSES and the rate of cognitive change and no association between late-life NSES and cognitive function. Our findings suggest that midlife NSES are relevant to late-life cognitive function, particularly for Black individuals. Incorporating a life-course perspective is crucial to understanding racial disparities in cognitive outcomes.

